# Aggregation of Disordered Proteins Associated with Neurodegeneration

**DOI:** 10.3390/ijms24043380

**Published:** 2023-02-08

**Authors:** Phoebe S. Tsoi, My Diem Quan, Josephine C. Ferreon, Allan Chris M. Ferreon

**Affiliations:** Department of Pharmacology and Chemical Biology, Baylor College of Medicine, Houston, TX 77030, USA

**Keywords:** neurodegenerative diseases, intrinsically disordered proteins, Aβ, tau, α-synuclein, TDP-43, AlphaFold, biomolecular condensates, liquid–liquid phase separation, LLPS

## Abstract

Cellular deposition of protein aggregates, one of the hallmarks of neurodegeneration, disrupts cellular functions and leads to neuronal death. Mutations, posttranslational modifications, and truncations are common molecular underpinnings in the formation of aberrant protein conformations that seed aggregation. The major proteins involved in neurodegeneration include amyloid beta (Aβ) and tau in Alzheimer’s disease, α-synuclein in Parkinson’s disease, and TAR DNA-binding protein (TDP-43) in amyotrophic lateral sclerosis (ALS). These proteins are described as intrinsically disordered and possess enhanced ability to partition into biomolecular condensates. In this review, we discuss the role of protein misfolding and aggregation in neurodegenerative diseases, specifically highlighting implications of changes to the primary/secondary (mutations, posttranslational modifications, and truncations) and the quaternary/supramolecular (oligomerization and condensation) structural landscapes for the four aforementioned proteins. Understanding these aggregation mechanisms provides insights into neurodegenerative diseases and their common underlying molecular pathology.

## 1. Introduction

Neurodegenerative diseases (NDs) are characterized by the progressive damage and dysfunction of neuronal cells. Despite exhibiting diverse clinical symptoms, these diseases share many common pathologic features. ND pathogenesis is thought to involve protein misfolding and aggregation within specific brain regions, which trigger neuroinflammation and oxidative stress at sites of injury, subsequently leading to degeneration of neural tissues. Molecular mechanisms responsible for the initial protein misfolding and the change from functional protein to pathologic aggregates have been a major subject of research in recent years. 

NDs are classified according to the clinical symptoms and the major component(s) of protein deposits found in affected brain regions [1]. A vast majority of these diseases are associated with a class of proteins known as intrinsically disordered proteins, or IDPs. These proteins can be entirely unstructured or hybrids of structured domains and long stretches of intrinsically disordered regions (IDRs). IDRs are primarily composed of polar or charged amino acids, lacking sufficient hydrophobic residues that can mediate cooperative folding [2]. IDPs exist in a dynamic equilibrium of multiple conformational states of varying degrees of folding under physiological conditions [3,4,5]. The conformations adopted are largely affected by factors such as amino acid sequence, embedded motifs, and charge distribution/arrangement [6,7,8].

**Figure 1:**
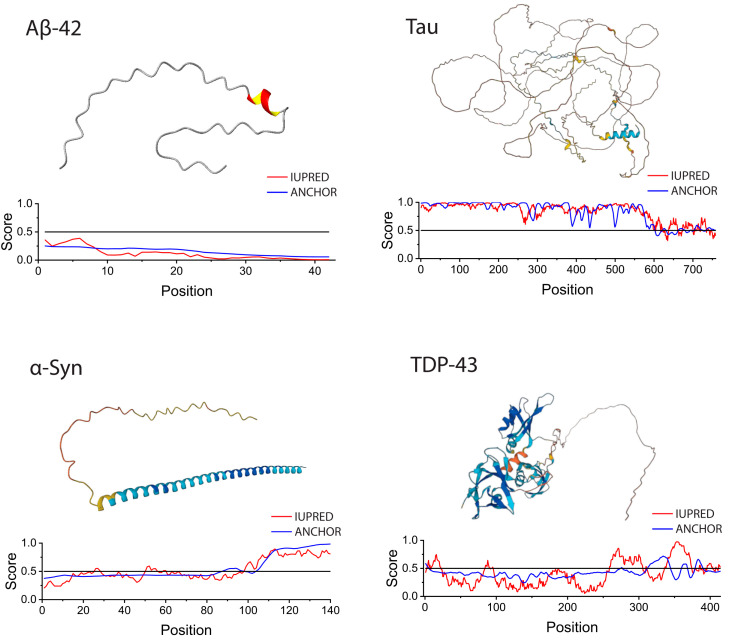
Predicted structure and disorder of Aβ, tau, α-syn, and TDP-43. Structures were predicted using AlphaFold [26], and calculations for disorder were determined using IUPRED2 [27,28,29,30]. IUPRED2 provides IUPred and ANCHOR scores for disordered proteins. ANCHOR predicts disorder based on amino acid sequence, identifying potential binding sites that are disordered in isolation, while IUPred predicts disorder based on energy estimation. IUPred scores from 0 to 0.5 are considered ordered while scores from 0.5 to 1 are considered disordered. Aβ-42 (human) was chosen as the representative peptide for Aβ. Tau (human, UniProt ID: P10636) is highly disordered with a C-terminal helical region. α-Syn (human, UniProt ID: P37840) has a disordered C-terminus and α-helical N-terminus. TDP-43 (human, UniProt ID: Q13148) contains a long unstructured C-terminus. (Note that these are predicted structures only and may not represent the major physiological protein conformations. For example, α-syn has been shown to adopt more random coil-like structures in physiological buffer conditions, with the elongated helical structure predicted using AlphaFold only favored in the presence of ligands such as membrane vesicles [31,32,33]).

IDPs are highly prevalent in many proteomes, including that of humans, and play important roles in cellular processes such as the regulation of transcription and translation [9,10], cell cycle control [11,12], and cell signaling [12,13]. Changes in the cellular milieu and/or mutation(s) in IDPs can disrupt normal protein functions, resulting in misfolding and aggregation/fibrillation [14,15]. Misfolded proteins can serve as conformational switches and/or seeds that proceed to self-propagation [16,17,18,19], taking on prion-like properties and causing cellular stress and damage [20,21]. Misfolded proteins can also cross-seed and induce other proteins to aggregate [22,23,24,25]. IDPs associated with common neurodegenerative diseases include amyloid beta (Aβ) and tau for Alzheimer’s disease (AD), α-synuclein (α-syn) for Parkinson’s disease (PD), and TAR DNA-binding protein (TDP-43) for amyotrophic lateral sclerosis (ALS) (Figure 1).

Aβ is a small, 39–43 amino acid peptide heavily implicated in AD. The peptide was identified to be the main component of neocortical plaques [34], a pathological hallmark of AD [35]. AlphaFold predicts Aβ-42 to be a primarily disordered peptide with a small helical region, whereas IUPred assigned a low disorder score. Experimentally, Aβ-42 exists as a mixture of random coils, α-helixes, and β-sheets, and specific conformations are favored based on environmental conditions [36,37], suggesting that Aβ-42 can be induced to favor conformations that are more aggregation prone. Tau is a microtubule-associated protein that exists as various isoforms with varying numbers of microtubule binding domains and 29-amino-acid-long inserts [38]. The isoform shown in Figure 1 contains three microtubule-binding repeats (tau-3R) and exhibits high disorder according to both AlphaFold and IUPred predictions. Tau is a major component of neurofibrillary tangles (NFTs) in AD [39]. α-Syn is a 140 amino acid protein whose mutated forms were first discovered in relation to PD [40]. α-Syn encodes a helical N-terminus, a non-amyloidal component domain (NAC), and an acidic, disordered C-terminal domain. TDP-43 is a 414 amino acid protein implicated in over 90% of ALS cases [41,42]. It contains a structured N-terminal domain, two RNA recognition motifs (RRMs), and a disordered low complexity C-terminal domain (LCD). 

**Figure 2:**
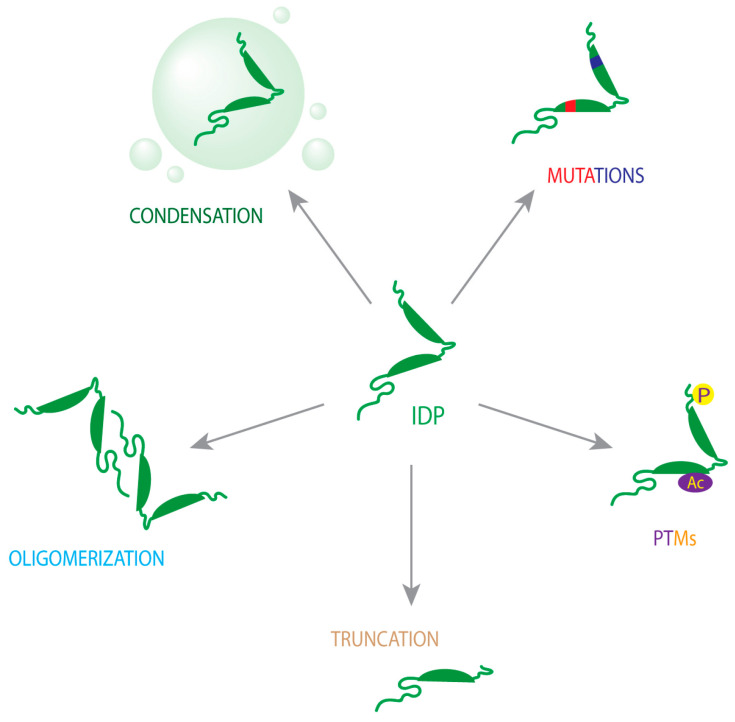
Molecular underpinnings of ND-linked IDP misfolding and aggregation.

The four aforementioned proteins undergo misfolding from their native states to form β-sheet-rich structures ranging from small oligomers to large fibrillar aggregates in diseased brains [43,44]. This review describes the molecular precursors responsible for misfolding and aggregation of the aforementioned ND-associated IDPs, summarized in Figure 2. 

## 2. Mutation

AD, PD, and ALS occur in both sporadic and familial forms. The majority of ND cases are sporadic, constituting approximately 97% of AD cases [45], 85% of PD cases [46], and 90–95% of ALS cases [47]. The remaining familial occurrences are caused by inheritable mutations, which in disease-related proteins generally exhibit similar phenotypes: increased aggregation propensities [48,49,50], altered protein populations [51,52,53] and proteasomal regulation [51,54], and increased cytotoxicity in model organisms [55,56,57]. 

Many Aβ mutations are located on the APP gene, which encodes for the amyloid precursor protein (APP) [58]. Most of the pathogenic mutations occur in a section of the APP gene that encodes the proteolytic sites of β- and γ-secretases, which result in an overall increased generation of Aβ [54,59]. Other APP mutations can affect the cleavage process from APP to Aβ and generate truncated products exhibiting differential aggregation propensities [52]. In particular, the Arctic APP mutant (APP E693G) displays high levels of protofibrils as well as cognitive defects in mice [55,60,61]. 

Tau protein is encoded by the MAPT gene, with both exonic and intronic mutations identified in tauopathies. Mutations generally promote tau aggregation by altering the ratio of tau containing three tubulin-binding repeats (tau-3R) to four tubulin-binding repeats (tau-4R), otherwise known as the 3R:4R tau ratio [48,49]. Tau-3R and tau-4R isoforms exist in a one-to-one ratio in most regions of the brain; deviations from this ratio characterize tauopathies [38]. Increased tau-4R expression has been found to promote tau phosphorylation and oligomerization and induce behavioral abnormalities in a mouse model expressing human tau [56]. Mutations also induce tau fragmentation and enhance tau hyperphosphorylation [51,62]. Extensively studied tau mutants include G272V, P301L, V337M, and R406W [63,64,65,66]; however, it should be noted that these mutants are associated with frontotemporal dementia (FTD), and not AD. No MAPT mutations have been associated with AD so far, suggesting that mechanisms underlying tau aggregation in AD may be different from those involved in other tauopathies caused by MAPT mutations.

α-Syn was first implicated in NDs when the A53T mutation was identified in autosomal dominant PD [40]. Autosomal dominant forms of PD are associated with N-terminal missense mutations, such as A53T, A53E, A30P, and E46K. [67,68,69]. Mutants A53T and A30P of α-syn have been shown to be structurally defective for membrane binding [31,32,70,71] and exhibit enhanced self-aggregation propensity and kinetics [72]. Mutant A30P α-syn has also been shown to exhibit 2-state folding thermodynamics, compared to the 3-state folding behavior of wild-type α-syn [71]. Wild-type α-syn is capable of assembling into two types of dimers, with one dimeric form more favored than the other [73]. Mutants A53T, A30P, and E46K have been demonstrated to promote dimerization and enhance the formation of the less favored dimeric structure [73]. The structural heterogeneity of α-syn dimers is suggested to indicate different aggregation pathways. More recently discovered mutations, A18T and A29S, were found to aggregate faster than wild-type α-syn, with the A18T mutant having faster aggregation kinetics compared to A29S [74]. 

Numerous mutations in the TARDBP gene have been identified as being associated with ALS. These TDP-43 mutations can increase aggregation propensity, enhance cytoplasmic mislocalization, and alter protein stability [50,75]. Most ALS-associated mutations appear in exon 6 of the TARDBP gene, which encodes for the intrinsically disordered C-terminal region of TDP-43. The most well-studied TDP-43 mutations include A315T, Q331K, M337V, and D169G, for which several ALS disease models have also been established [50]. Recombinantly expressed TDP-43 containing ALS-linked mutations were found to have increased aggregation in vitro and promoted cytotoxicity in yeast cells [57]. Peptides from the TDP-43 amyloidogenic core region (residues 286–366) containing ALS-associated mutations also form amyloid-like fibrils [76,77]. TDP-43 A315T mutant has been found to form amyloid fibrils in vitro and cause cell death when added to cultured neuronal cells [77]. Additional information regarding mutations, as well as reviews that discuss the role of mutations in NDs in-depth, can be found in Table 1.

## 3. Posttranslational Modification

Posttranslational modifications (PTMs) are frequently used to regulate IDP function, localization, and turnover [85,86]. Aberrant PTMs disrupt Aβ, tau, α-syn, and TDP-43 functions and are linked to neurodegeneration. The mechanisms by which PTM leads to disease pathology are largely dependent on the type of PTM and the protein involved. In general, however, PTMs, such as phosphorylation and acetylation, alter charge properties, affect binding interactions [87], folding and conformational stability [88], and oligomerization states [89], all of which exhibit the ability to modulate aggregation. Pathologic PTMs include phosphorylation, ubiquitination, acetylation, and glycosylation.

PTMs of Aβ have been demonstrated to increase its aggregation rate. Phosphorylation of residue S8 increases the stability of the β-sheet conformation of Aβ [90,91], and phosphorylated Aβ have been detected in the brains of transgenic mice and AD patients [88]. In Drosophila, phosphorylated Aβ induced higher toxicity compared to non-phosphorylated Aβ [90]. In addition to phosphorylation, glycation also stimulates amyloid aggregation. Aβ in amyloid deposits are glycated [92]; advanced glycation of Aβ can seed and accelerate aggregation of soluble Aβ peptide [93,94]. Cerebral spinal fluid of AD patients contain abnormally O-glycosylated Aβ peptides of 15–17 residue size [95], suggesting that glycosylation may play a role in Aβ clearance [96]. 

Tau hyperphosphorylation is responsible for its loss of physiological functions, gain in toxicity, and aggregation in the form of NFTs. Specifically, residues S396, S404, and S422 have been found abnormally hyperphosphorylated in diseased brains [97], and it has been suggested that phosphorylation at these residues may influence tau aggregation [98]. Hyperphosphorylation has also been shown to impair microtubule binding [87], which could lead to microtubule destabilization and compromised cytoskeletal integrity. Tau acetylation was first recognized in ND mouse models [99,100]. Acetylation of K280 weakens the binding of tau to negatively charged microtubules, potentially destabilizing microtubule networks [101,102]. Ubiquitin has been identified in tau inclusions extracted from the brains of tauopathy patients; however, the role of ubiquitination in ND is not yet known. While ubiquitin is a component of tau aggregates found in the brains of AD patients, tau phosphorylation precedes its ubiquitination in the NFTs of AD patients [103,104]. This suggests that ubiquitin may be linked to tau after the formation of the fibrillar inclusions. However, other groups have reported that both mono- and polyubiquitination contribute to the formation of insoluble protein inclusions present in neurodegenerative diseases [105,106], and that tau ubiquitination in cell cultures increases aggregation [107].

Addition of side chain modifiers has also been shown to modulate α-syn toxicity. Physiological levels of phosphorylated α-syn are relatively low; however, threonine, serine, and tyrosine hyperphosphorylation are commonly found in pathologically aggregated α-syn [108,109]. Most phosphorylated residues are located in the C-terminus of α-syn, which is thought to be involved in α-syn pathology. S129, in particular, is phosphorylated in >90% of PD patients and is used as a pathological marker [108]. Ubiquitinated α-syn is often present in inclusions of PD patients in conjunction with phosphorylation [110]. Sumoylation [111,112], nitration [113], and glycosylation [114] of α-syn have also been observed in association with α-syn toxicity.

The two most pathologically significant PTMs in TDP-43 are phosphorylation and ubiquitination. TDP-43 phosphorylation is a signature of ALS pathology; S409/S410 phosphorylation, in particular, are distinctly observed in ALS patients [115]. Phosphorylation is associated with cytoplasmic mislocalization and aggregation of TDP-43 in neurons [116,117]. TDP-43 has also been found in the ubiquitinated state in ALS brain inclusions [42]. Ubiquitination facilitates TDP-43 cytoplasmic accumulation into inclusions without any detectable evidence of its degradation [118,119]. Additionally, acetylation may serve a pivotal role in mediating TDP-43 function and dysfunction [120].

PTM is an ever-expanding field of research. Although the PTMs most discussed in-depth in this review were phosphorylation and ubiquitination, many other PTMs affect these proteins and other disease-related IDPs. Table 2 provides references to additional resources that discuss the roles of PTM addition for each protein. Further investigation of other covalent modifications is warranted to build a more comprehensive picture of how the cells regulate behaviors of ND-associated proteins and how aberrant regulations can be rescued.

## 4. Truncation

Protein truncation is one of the most common pathological modifications of IDPs. Truncations in IDPs can alleviate steric hindrance involved with protein folding, inducing structural changes that can lead to protein misfolding [126]. The truncated forms exhibiting perturbed aggregation behavior can act as seeds for nucleation and partake in self-assembly that results in the formation of insoluble structures. Moreover, truncation is known to impede vital functions of IDPs, leading to both loss-of-function and toxic gain-of-function. A plethora of studies have demonstrated that truncated forms of neuronal IDPs are the driving force in various neurodegenerative proteinopathies [51,127,128]. 

Aβ peptides are generated by the cleavage of APP via β- and γ-secretases [129]. In nonneuronal cells, the majority of APP proteins are initially cleaved by α-secretase. This nonamyloidogenic cleavage occurs within the Aβ domain and prevents the production of Aβ [130]. In contrast, through the amyloidogenic pathway, β-secretase mediates the initial cleavage of APP, which is subsequently processed by γ-secretase to produce Aβ peptides [131,132]. The amyloidogenic cleavage occurs within the transmembrane domain of APP and generates C-terminally truncated peptides of various sizes, ranging from 38 to 42 amino acids. The levels of generated peptides are used to distinguish AD from other NDs [133,134]. The Aβ species most strongly implicated in AD is Aβ-42. Aβ-42 exhibits enhanced aggregation propensity compared to other Aβ peptides [135]. Although healthy individuals can also generate Aβ peptides, higher levels of Aβ-42 are detected in AD patients’ brain samples [133,136], and AD patients have been observed to generate longer Aβ forms compared to unafflicted individuals [127]. 

Truncated tau species are derived from proteolytic processing via proteases, of which caspases and calpains are of particular interest. Specific cleavage products of caspases −2, −3, and −6 have been linked to AD. A truncated form of tau generated by caspase-2 cleavage at D314 has been found in AD brains [51,137]. Caspase-3 cleavage at D421 generates a tau-421 species; elevated levels of caspase-3 and tau-421 have been observed in AD [51,138]. Tau-421 colocalizes with NFTs in human AD brain and correlates with NFT formation and cognitive impairment in aged mice [139]. Tau can also be cleaved by caspase-6 to produce tau-13 and tau-402 truncations. Active caspase-6 and tau-402 were observed in NFTs and neuritic plaques in the AD brain [140]. In addition, tau-402 levels in cerebrospinal fluid correlate with impaired cognitive performance in AD patients [141]. Calpains are calcium-dependent cysteine proteases, and calpain-mediated tau cleavage generates several truncated tau isoforms such as tau-45–230 and tau-243–441 [142,143]. Increased levels of tau-45–230 have been identified in AD brain samples, and elevated tau-243–441 levels are observed in transgenic tau Tg601 mouse model [143]. 

α-Syn inclusions in human brain contain C-terminally truncated α-syn protein, which may be generated by proteasome degradation or calpain cleavage [144,145]. C-terminally truncated α-syn fibrillizes in vitro [146], and mice expressing this α-syn species in dopaminergic neurons demonstrate neuronal aggregates with either granular or fibrillar morphologies [147]. Caspase cleavage of α-syn generates 1–121 α-syn. This truncated peptide assembles into fibrils and demonstrates prion-like seeding [128,148].

The C-terminal region of TDP-43 is highly disordered and comprises a glycine-rich region. Highly cytotoxic C-terminal fragments of sizes 25–35 kDa that are produced from aberrant caspase cleavage of TDP-43 are the prominent species found in the inclusion bodies identified from ALS-affected brains [149,150]. The C-terminal region of TDP-43 also contains a short, highly dynamic and unstable helix–turn–helix region in residues 311–360. Peptides containing this region form amyloid-like fibrils in vitro, which can exhibit prion-like toxicity in cells [76,151]. Table 3 provides additional resources that discuss different protein truncations and their roles in NDs.

## 5. Toxic Oligomerization

Significant evidence suggests that smaller, soluble misfolded oligomers may be the true cause of neurodegeneration [155,156,157]. Misfolded oligomers are a group of species that exist in a range of sizes, from dimers to protofibrillar structures [158,159,160]. These oligomeric species are highly dynamic and exist in equilibrium with monomers and fibrils. Some oligomers may be intermediates for amyloid fibril formation while others might be terminal, off-pathway products [160,161]. The heterogeneity, thermodynamic interconversion between species, and aggregation propensity of these oligomers have made it very difficult to obtain high-resolution structural information, as well as to determine which are the most relevant oligomeric structures for disease [157,159].

The Aβ oligomer hypothesis was introduced in 1998 and suggested that the damage found in AD patient brains is caused by oligomeric species [162]. Since then, oligomeric species of various sizes have been identified in human brains and in brains from APP transgenic mice, although the characterization of these oligomers has been hindered by their metastability and heterogeneity [159]. Oligomeric species that are ‘on-pathway’ and ‘off-pathway’ to fibril formation have been observed [163]. Some oligomers (>50 kDa) identified by mass spectrometry are toxic and off-pathway of amyloid formation [164,165] while other, smaller species (<50 kDa) that react with anti-fibril antibodies readily form fibrils and are less associated with toxicity [166,167]. Aβ can also assemble into intermediate structures known as protofibrils, which are large (>100 kDa) on-pathway species for fibrillation [168]. These Aβ protofibrillar species, which have been shown to induce neurotoxicity in rat cortical neurons [168] and impair cognitive and behavioral functions (such as spatial–temporal pattern separation and learning in mice [169]), are associated with inflammatory responses, and have been detected in AD brains [170]. 

Oligomeric tau may also be the toxic species in tauopathies. Hyperphosphorylated tau assembles into oligomers prior to NFT formation. Hyperphosphorylated tau monomers have lowered affinity to microtubules and increased affinity for other tau monomers to form oligomeric tau that is detergent-soluble. These tau oligomers potentiate neuronal damage, leading to neurodegeneration and traumatic brain injury [89,171,172]. In AD brain samples, tau oligomers were found at a fourfold greater concentration compared to healthy control samples [173]. When the oligomer grows, it adapts a β-sheet structure and transforms into a detergent-insoluble aggregate with granular appearance, which elongates into tau fibrils and ultimately forms NFTs [174]. This process suggests that tau oligomers may be involved in neuronal dysfunction prior to NFT formation [175].

α-Syn matures from monomer to amyloid fibrils rich in β-sheets through several intermediate oligomeric species [176,177]. Single-molecule studies of α-syn have identified conformations that can initiate pathologic aggregation [31,71,178]. Findings have suggested that certain α-syn oligomers or protofibrils may be toxic [179,180]. The initial observation that the mutant A30P α-syn monomers were consumed more rapidly but fibrillized more slowly than WT α-syn suggests that the oligomeric intermediary species may be pathologic, rather than the fibrillar forms [176,177]. Direct in vivo data supporting the toxic protofibril hypothesis are still relatively limited, and most of the evidence is circumstantial.

TDP-43 oligomerization and its potential neurotoxic properties have also been studied. In normal brains, TDP-43 exist as dimers in the nucleus of neurons [181,182]. However, there are reports of pathologic TDP-43 oligomers, which may be structurally distinct from the nuclear oligomers. The N-terminal domain regulates dimerization [181,183], and the N-terminal region (residues 3–183) acts as an intermolecular interacting domain for an 86 kDa species that was observed in an immunoblot of extracts from deceased ALS patient brains [184]. Full-length TDP-43, not only certain domains, has also been shown to form stable, spherical cytotoxic oligomers in neuronal cells [185]. These TDP-43 oligomers can also cross-seed Aβ-42 peptide, demonstrating a structural conversion that can occur among common amyloid species [185,186]. Furthermore, such TDP-43 oligomeric aggregates were detected in brain sections of TDP-43 mouse models as well as ND patients [185]. 

## 6. Biomolecular Condensation

Biomolecular condensates (BMCs) include membraneless organelles, which are small compartments that are not encased in lipid membranes. BMC formation can be driven by liquid–liquid phase separation (LLPS)—a process by which a solution demixes into two liquid states: one phase concentrated in macromolecules and another dilute phase. [187,188,189]. It should be noted that while IDPs typically show increased tendency to phase separate, disorder is not a prerequisite for phase separation. Structured domains can also be induced to phase separate [190]. BMCs congregate and concentrate proteins and nucleic acids that are involved in diverse processes, including RNA metabolism, DNA damage response, and signal transduction. Although these condensates play many physiological roles [191,192], their aberrant behaviors may be associated with disease, especially neurodegeneration [193,194,195].

In the case of Aβ, recruitment into BMCs may actually be protective. A recent study by Kuffner et al. demonstrated that Aβ-42 was recruited into a liquid droplet along with other scaffold proteins of membraneless organelles. Although Aβ-42 concentration increased in the sequestered phase, its aggregation was inhibited [196]. This result suggests that another role of BMCs is to sequester aggregation-prone proteins as a protective measure.

Tau phase separation plays a role in its biological and pathological functions. Tau decreases the critical concentration for tubulin assembly [197]. In the presence of tau condensates, tubulin assembly is further facilitated, indicating that tau condensates may act as nucleation sites for microtubules [191]. Phase separation may also initiate tau aggregation, as phosphorylated tau has higher phase separation propensity [193,198]. Tau phase separation has also been shown to be inhibited by acetylation [199]. These studies suggest that tau BMCs serve a functional role that can be modulated by PTMs, although further studies are required to fully elucidate the regulation of BMCs.

α-Syn has been shown to phase separate in vitro in the presence of molecular crowding agents, although at high critical concentrations and after prolonged incubation [200]. Mutations and PTMs significantly lower this critical concentration and decrease the time necessary to form droplets [194]. These observations suggest that the phase separation properties of α-syn are more closely related to its aggregation.

TDP-43 BMCs have both functional and dysfunctional relevance. TDP-43 is a component of stress granules, transient repositories of proteins and RNAs that form when cells are exposed to internal or external stressors [192,195]. TDP-43 is also found in insoluble cytoplasmic inclusions that are hypothesized to originate from stress granules [195]. In vitro studies of TDP-43 have shown that phase separation facilitates aggregation of the unstructured C-terminus [201], and that fibrillation and phase separation can be decoupled [202]. ALS-relevant TDP-43 mutants disrupt phase separation and enhance aggregation, though with irregular morphology [203]. 

## 7. Conclusions

With the emergence of IDP roles in vital cell functions, as well as their implications in various diseases, the field of IDP research has been rapidly expanding within recent decades. This review sought to summarize the molecular mechanisms that underlie the major IDPs associated with the most common neurodegenerative diseases. Mutations, aberrant PTMs, and truncations contribute to the pathologic properties of ND-associated proteins Aβ and tau, α-syn, and TDP-43, respectively, associated with AD, PD, and ALS. The capability of each protein to assemble into cytotoxic oligomeric species that may evolve into the aggregated hallmarks of their respective diseases as well as the functional and dysfunctional duality of their biomolecular condensates demonstrate that the pathway from functional to pathological is not straightforward, rather winding with multiple avenues for regulation and therapy. While this review is primarily focused on four specific proteins, these trends can be observed in other IDPs associated with neurodegenerative diseases such as TIA-1, hnRNP, FUS, ataxin, and SOD1. Continued research in this field will allow us to further understand the pathology of neurodegenerative diseases and develop effective therapeutic strategies.

## Figures and Tables

**Table 1:** Mutations associated with NDs.

Protein	Mutation	Citation
Aβ	E693G	[55,60,61]
		* [78]
tau	G272V	[65,66,79]
	P301L	[64,65,66,79]
	V279M	[64]
	V337M	[64,79]
	R406W	[65,79]
		* [80]
α-Syn	A53T	[40,67,68]
	A53E	[81]
	A30P	[67,69]
	E46K	[67]
		* [82]
TDP-43	A315T	[83]
	Q331K	[75]
	M337V	[75]
	D169G	[83,84]
		* [50]

* In-depth review and additional mutations.

**Table 2:** PTMs associated with NDs.

Protein	PTM	Residue	Citation
Aβ	Phosphorylation	S8	[90]
	Glycation	N-term/K residues	[92]
	Glycosylation	Y10	[121]
			* [122]
tau	Phosphorylation	S396	[97]
		S404	[97]
		S422	[97]
	Acetylation	K280	[99]
	Ubiquitination	K48	[103]
		K63	[105]
			* [123]
α-Syn	Phosphorylation	S129	[108]
	Ubiquitination	K6	[124]
		K12	[124]
		K23	[124]
			* [124]
TDP-43	Phosphorylation	S409	[115]
		S410	[115]
	Ubiquitination	K48	[125]
		K63	[125]
			* [125]

* In-depth review with information about additional PTMs.

**Table 3:** Truncations associated with NDs.

Protein	Truncation	Citation
Aβ	Aβ-38	[133]
	Aβ-40	[133]
	Aβ-42	[133,134]
		* [121]
tau	tau-314	[51,137]
	tau-421	[51,138]
	tau-13	[140]
	tau-402	[140]
	tau-45–230	[141]
	tau-243–441	[143]
		* [152]
α-Syn	1–121	[128,148]
		* [53]
TDP-43	TDP-90–414	[149]
	TDP-220–414	[149,153]
		* [154]

* In-depth review and information about additional truncations.

## Data Availability

Not applicable.
